# THE CREATION OF THE FIRST INTERNATIONAL GSA STUDENT CHAPTER IN PORTUGAL

**DOI:** 10.1093/geroni/igad104.0605

**Published:** 2023-12-21

**Authors:** Giuliana Casanova, Lígia Passos, Edna Rodrigues, Oscar Ribeiro

**Affiliations:** University of Aveiro, Aveiro, Aveiro, Portugal; University of Aveiro, Aveiro, Aveiro, Portugal; School of Medicine and Biomedical Sciences, Porto, Porto, Portugal; University of Aveiro, Aveiro, Aveiro, Portugal

## Abstract

The Gerontological Society of America (GSA) Student Chapter Pilot Program provides a platform for students in all academic levels to exchange academic ideas, network with industry professionals, and share scientific and educational experiences. Developing an international student chapter based at the University of Aveiro in Portugal was a challenging but fullfiling task, accomplished through the dedication and perseverance of its members. This student chapter, created by and for students, aims to serve as an opportunity for networking, education, research, and community work. We aspire to create a bridge between Portugal and the United States, promoting educational and research opportunities for students and increasing awareness in the field of aging. By sharing the challenges and rewards we encountered during the creation of this chapter, we hope to inspire other universities worldwide to do the same and build an even larger international network. Through this network, students working with older adults can find common ground, share ideas, create connections, and develop new ways to enhance the field of Gerontology worldwide. The GSA student chapter is a unique opportunity that enables students to connect, share knowledge and contribute to the field of Gerontology. Our hope is that this model will be replicated globally, fostering collaboration and advancing our understanding of aging in all corners of the world.

